# Myasthenia Gravis Related to Small Cell Lung Carcinoma

**DOI:** 10.7759/cureus.13889

**Published:** 2021-03-14

**Authors:** Laura López-Viñas, Esmeralda Rocío-Martín, Salvador Delis-Gómez, Rybel Wix-Ramos

**Affiliations:** 1 Clinical Neurophysiology Department, Fundación Jiménez Díaz University Hospital, Madrid, ESP; 2 Clinical Neurophysiology Department, La Princesa University Hospital, Madrid, ESP; 3 Neurology Department, University Hospital of Araba, Vitoria-Gasteiz, ESP

**Keywords:** electromyography, clinical neurophysiology, myasthenia gravis, paraneoplastic syndrome, small cell lung carcinoma

## Abstract

Myasthenia gravis is a neuromuscular disease that causes weakness in skeletal muscles because of the presence of acetylcholine receptor antibodies. These antibodies produce a compromise in the end-plate potential, reducing the safety factor for effective synaptic transmission. Clinically, this manifests as muscle weakness and, in severe cases, respiratory failure.

There is widespread knowledge about the association between small cell lung carcinoma and Lambert- Eaton myasthenic syndrome, but not with other neuromuscular disorders, such as myasthenia gravis.

We present a patient with small cell lung carcinoma who presented weakness affecting the proximal muscles over the last three years, and electromyography findings suggesting myasthenia gravis. After this electrodiagnosis, analytical tests showed an increase in anti-acetylcholine receptor antibodies.

Given these findings, we can affirm that neurophysiological tests provide a significant value in diagnosing myasthenia gravis, as anti-acetylcholine receptor antibodies were negative at the moment of the electromyography’s performance. Likewise, it is essential to consider a paraneoplastic syndrome in this type of carcinoma.

## Introduction

Myasthenia gravis (MG) is a disease in which neuromuscular transmission is affected because of the action of the antibodies at the postsynaptic level, causing weakness of skeletal muscles [1,2]. Small cell lung carcinoma (SCLC) is usually associated with different paraneoplastic syndromes that affect the neuromuscular junction.

Lambert-Eaton myasthenic syndrome (LEMS) is the entity that has been most frequently associated with SCLC. MG was diagnosed in a few cases of patients with this type of carcinoma. In other previous studies, SCLC was diagnosed after generalized weakness. Along with the performing of neurophysiological studies (repetitive nerve stimulation [RNS] and single-fibre electromyography [EMG]), MG was confirmed [3,4]. A theory supporting this relation is the invasion of the malignant cells of this type of cancer into the mediastinum's lymph nodes, developing the typical clinic of MG [5].

## Case presentation

The patient is a 67-year-old woman who complained of loss of strength, affecting predominantly the proximal muscles, in both the upper and lower limbs, over the last three years. The patient reported recently marked difficulties in climbing stairs and walking. Symptoms presented fluctuated initially, becoming continuous at the time of assessment. Bulbar or respiratory symptoms did not accompany them.

Her past medical history was remarkable for the presence of small cell carcinoma of the lung in treatment with carboplatin and etoposide; the patient has been a smoker from age 16 and is a moderate alcohol drinker. On neurological examination, manual muscle strength testing was 5-/5 in the proximal upper limbs and 4+/5 in the proximal lower limbs. The sensation was preserved to all modalities. The bicipital, patellar and Achilles deep tendon reflexes were absent bilaterally.

Neurophysiologists performed electroneurography, EMG, RNS, and single-fibre EMG. Electroneurography for nerve conduction studies (NCS) revealed normal motor and sensory conduction velocities and expected characteristics of the evoked potentials, helping us rule out polyneuropathy secondary to chemotherapy treatment. During the needle EMG, they studied the right vastus lateralis muscle, which showed no evidence of abnormalities, ruling out a myopathy possibility.

**Figure 1:**
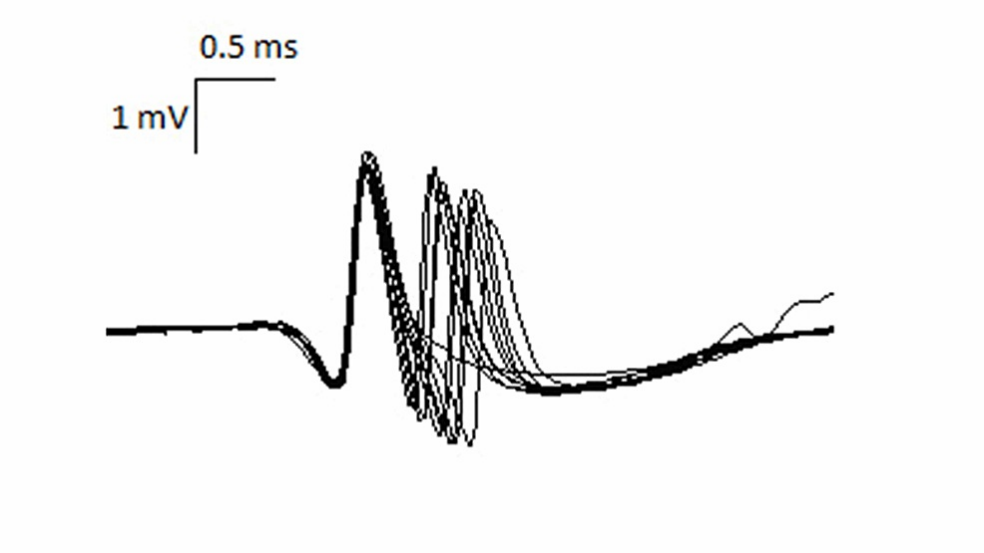
Single-fibre EMG of the right vastus lateralis muscle showing the presence of an increased jitter and blocking. EMG: electromyography

For the RNS test, they studied the right median nerve. The responses were recorded using electrodes applied over the abductor pollicis brevis muscle. It demonstrated a significant drop (>10%) in the amplitude of the fourth potential to the first one. Moving next to the single-fibre EMG, the right vastus lateralis muscle was studied, which showed the presence of an increased jitter in four of the six pairs, with an increase of the average jitter value. In one of the pairs analyzed, there was a block (Figure 1).

These findings were consistent with an alteration of the neuromuscular transmission at the postsynaptic motor end-plate level, which is compatible with MG.

Her laboratory studies showed that anti-acetylcholine receptor (anti-AchR) antibodies (Ab) testing was negative (0.38 IU/mL). In the presence of clinical and neurophysiological data suggestive of MG, physicians repeated the laboratory workup (which includes anti-AchR Ab, anti-striated muscle Ab and anti-MuSK Ab), highlighting an anti-AchR Ab value of 0.75 IU/mL (a grey area of positivity). After one year, a control analysis showed an anti-AchR Ab value of 1.15 IU/mL (positive). Also, a chest radiograph and computerised tomography (CT) were performed, not observing the presence of an associated thymoma.

## Discussion

MG is an autoimmune disease of the neuromuscular junction with the production of anti-AchR Ab located at the skeletal muscles. These receptors are depleted by inducing AchR internalization, resulting in smaller end-plate potentials. Accordingly, the most frequent clinical presentation is muscle weakness, with the possible development of respiratory failure. Its diagnosis requires clinical history, examination, serological tests for anti-AchR Ab, and a chest CT to search for thymoma and neurophysiological tests. The treatment depends on the disease stage and includes plasmapheresis, thymus removal, steroids, immunomodulators and anticholinesterase drugs [6,7].

Neuromuscular weakness in SCLC patients is prevalent, and one must consider a variety of differentials when evaluating it. Some possibilities include weakness secondary to the disease itself or chemotherapy-related weakness. Here we present a case highlighting de novo MG in the setting of SCLC.

Assessing the possibility of a myasthenic syndrome secondary to the SCLC presented by the patient, the relationship of the laboratory values of the anti-AchR Ab with this disease has been widely described since the receptor subunit α3-nicotinic is neuronal and the human thymus is capable of expressing it, causing the appearance of MG [4]. Other cases described an initial suspicion of subacute polyneuropathy. Because of the normal results of the electroneurography and the presence of SCLC, physicians decided to perform other neurophysiological techniques, such as RNS and single-fibre EMG, which were suggestive of MG. Also, anti-AchR Ab were positive in those cases.

Another hypothesis related to the presence of MG in the context of SCLC is that cancer cells from SCLC express the α3-nicotinic subunit gene; when they cross-react with the muscle α1-nicotinic muscle receptor subunit, they could phenotypically express the symptoms suggestive of MG. Another hypothesis describes the presence of the squamous cell carcinoma (SCC)-37 and SCC-A9 cell lines in SCLC, which aberrantly expresses the nicotinic acetylcholine receptors and achieves a high desensitization rate, increasing the small transitory depolarizations occurring in MG [5,6].

It is essential to point out the role of neurophysiological tests in detecting this disease since there have been studies of patients with SCLC who presented MG and for whom the anti-AchR Ab were negative, such as in the above case. Also, other authors described neurophysiological techniques suggestive of MG as a marked decremental response in RNS specific to a postsynaptic neuromuscular disease previously to the results of anti-AchR Ab.

## Conclusions

One of the main paraneoplastic syndromes associated with SCLC is LEMS, although we should consider other neuromuscular diseases that are less prevalent, such as MG. Furthermore, electromyography could show findings suggesting MG previously to blood analysis.
